# Supplementary material for: Novel Bacillus-Infecting Bacteriophage B13—The Founding Member of the Proposed New Genus Bunatrivirus
Source: Viruses. 2022 Oct 19;14(10):2300. doi: 10.3390/v14102300 (PMC9610010; doi:10.3390/v14102300)
Supplement: Supplementary file 1 [file viruses-14-02300-s001.zip › viruses-1916611-supplementary.pdf]

Novel *Bacillus*-infecting Bacteriophage B13 – the Founding Member of the Proposed New Genus *Bunatrivirus*

Olesya A. Kazantseva [*], Emma G. Piligrimova, and Andrey M. Shadrin

Laboratory of Bacteriophage Biology, G. K. Skryabin Institute of Biochemistry and Physiology of Microorganisms, Pushchino Scientific Center for Biological Research of the Russian Academy of Sciences, Federal Research Center, Pushchino, Moscow Oblast, Russia

Supplementary Information:

[*] Correspondence: olesyakazantseva@bk.ru (O.A.K.); andrey2010s@gmail.com (A.M.S.)

Table S1. The host range of the B13 phage determined on 38 *Bacillus* strains.

№	Bacterial species	Strain	Source	Phage lysis
1	*B. cereus*	VKM B-13	VKM	-
2	*B. cereus*	VKM B-15	VKM	+
3	*B. cereus*	VKM B-370	VKM	+
4	*B. cereus*	VKM B-373	VKM	+
5	*B. cereus*	VKM B-374	VKM	+
6	*B. cereus*	VKM B-383	VKM	-
7	*B. cereus*	VKM B-445	VKM	+
8	*B. cereus*	VKM B-473	VKM	+
9	*B. cereus*	VKM B-491	VKM	+
10	*B. cereus*	VKM B-504^T	VKM	+
11	*B. cereus*	VKM B-681	VKM	+
12	*B. cereus*	VKM B-682	VKM	+
13	*B. cereus*	VKM B-683	VKM	+
14	*B. cereus*	VKM B-684	VKM	+
15	*B. cereus*	VKM B-686	VKM	-
16	*B. cereus*	VKM B-687	VKM	-
17	*B. cereus*	VKM B-688	VKM	+
18	*B. cereus*	VKM B-771	VKM	-
19	*B. cereus*	VKM B-810	VKM	-
20	*B. cereus*	VKM B-811	VKM	-
21	*B. cereus*	VKM B-812	VKM	-
22	*B. cereus*	ATCC 4342	ATCC	-
23	*B. cereus*	ATCC 14893	probiotic"Bactisubtil"[1]	-
24	*B. thuringiensis*	VKM B-83	VKM	+
25	*B. thuringiensis*	VKM B-84	VKM	+
26	*B. thuringiensis*	VKM B-85	VKM	+
27	*B. thuringiensis*	VKM B-440	VKM	-
28	*B. thuringiensis*	VKM B-443	VKM	-
29	*B. thuringiensis*	VKM B-446	VKM	-
30	*B. thuringiensis*	VKM B-447	VKM	-
31	*B. thuringiensis*	VKM B-450	VKM	+
32	*B. thuringiensis*	VKM B-453	VKM	+
33	*B. thuringiensis*	VKM B-454	VKM	+
34	*B. thuringiensis*	VKM B-1555	VKM	-
35	*B. thuringiensis*	VKM B-1557	VKM	-
36	*B. thuringiensis*	ATCC 35646	ATCC	+
37	*B. weihenstephanensis*	KBAB4	[2]	+
38	*B. flexus*		Laboratory collection	-

Source abbreviations: VKM: All-Russian Collection of Microorganisms; ATCC: American Type Culture Collection.

Table S2. Annotation of *Bacillus* phage B13.

ORF№	Start codon	Stop codon	Strand	Blast results		Conserved domains, Blast		Hhpred results	Annotation
				Name	E-val	Name, (region)	E-val	(Prob./E-val)	
1	76	438	+	TPA: MAG TPA: terminase small subunit [*Myoviridae* sp.]<br><br>TPA: MAG TPA: terminase small subunit [*Myoviridae* sp.]<br><br>TPA: MAG TPA: terminase small subunit [*Myoviridae* sp.]	2.0e-33<br><br>2.0e-33<br><br>5.0e-31	-	-	COG3747; COG3747; Phage terminase, small subunit [Mobilome: prophages, transposons] (99.22/1.70e-10)	terminase, small subunit
2	435	2135	+	terminase large subunit [*CaldiBacillus* phage CBP1]<br><br>TPA: MAG TPA: Large Terminase [*Siphoviridae* sp.]<br><br>TPA: MAG TPA: Large Terminase [*Siphoviridae* sp.]	0.0e+00<br><br>0.0e+00<br><br>0.0e+00	YmfN (52-561)<br><br>Terminase_1 (87-552)	1.47e-97<br><br>3.71e-76	P59217; TERL_BPSF5 Putative terminase large subunit (100.0/1.50e-58)<br><br>COG4626; YmfN; Phage terminase-like protein, large subunit, contains N-terminal HTH domain (100.0/4.60e-56)<br><br>O21870; TERL_BPLSK Terminase large subunit (100.0/2.00e-49)	terminase, large subunit
3	2149	3372	+	TPA: MAG TPA: portal protein [*Myoviridae* sp.]<br><br>portal protein [*Clostridium* phage phiCD506]<br><br>portal protein [*Clostridium* phage phiCDHM11]	0.0e+00<br><br>0.0e+00<br><br>0.0e+00	Phage_portal (41-361)	1.33e-64	Q6QGD5; PORTL_BPT5 Portal protein (100.0/5.00e-43)<br><br>P49859; PORTL_BPHK7 Portal protein (100.0/1.30e-40)	portal protein

								O21872; PORTL_BPLSK Probable portal protein (100.0/3.50e-40)	
4	3329	3913	+	capsid maturation protease [*PaeniBacillus* phage Dragolir]<br><br>prohead protease [*Bacillus* phage J5a]<br><br>prohead protease [*Bacillus* phage z1a]	2.0e-42<br><br>2.0e-41<br><br>4.0e-41	Peptidase_S78 (14-174)	2.53e-72	COG3740; COG3740; Phage head maturation protease (99.96/2.30e-27)<br><br>P49860; PRO_BPHK7 Prohead protease (99.93/7.40e-24)<br><br>D3WAC4; PRO_BPLP2 Probable capsid maturation protease (99.89/7.70e-21)	prohead protease
5	3930	5114	+	TPA: MAG TPA: major capsid protein [*Siphoviridae* sp.]<br><br>TPA: MAG TPA: major capsid protein [*Siphoviridae* sp.]<br><br>TPA: MAG TPA: major capsid protein [*Siphoviridae* sp.]	3.0e-165<br><br>2.0e-164<br><br>1.0e-159	major_cap_HK97(3-390)<br><br>Phage_capsid(124-389)<br><br>COG4653(88-394)	1.23e-68<br><br>1.64e-48<br><br>3.02e-10	COG4653; COG4653; Predicted phage phi-C31 gp36 major capsid-like protein (100.0/1.10e-35)<br><br>P49861; CAPSD_BPHK7 Major capsid protein (100.0/1.00e-33)<br><br>Q6QGD8; CAPSD_BPT5 Major capsid protein (100.0/3.60e-32)	major capsid protein
6	5130	5387	+	hp	hp	-	-	d1xp8a2; d.48.1.1 (A:283-341) RecA protein, C-terminal domain (90.72/4.60e-01)	hp
7	5384	5656	+	DNA packaging protein [*PaeniBacillus* phage PG1]<br><br>TPA: MAG TPA: head to tail adaptor [*Myoviridae* sp.]	1.0e-26<br><br>3.0e-24	put_DNA_pack (2-88)	5.14e-13	cd08054; gp6; Head-Tail Connector Protein gp6 of Bacteriophage HK97 and similar proteins (99.57/1.40e-14)	head completion protein

				TPA: MAG TPA: head to tail adaptor [*Siphoviridae* sp.]	3.0e-24			cd08051; gp6_gp15_like; Head-Tail Connector Proteins gp6 and gp15, and similar proteins. Members of this family include the prote(99.49/1.60e-13)<br><br>Q38584; HCP15_BPSPP Head completion protein gp15 (99.36/8.50e-12)	
8	5653	5952	+	TPA: MAG TPA: head closure knob [*Myoviridae* sp.]<br><br>TPA: MAG TPA: head closure knob [*Myoviridae* sp.]<br><br>TPA: MAG TPA: head closure knob [*Siphoviridae* sp.]	6.0e-21<br><br>5.0e-20<br><br>1.0e-19	gp16_SPP1 (2-96)	2.78e-08	COG5614; COG5614; Bacteriophage head-tail adaptor (99.87/1.20e-20)<br><br>O48446; HCP16_BPSPP Head completion protein gp16 (99.83/1.60e-18)<br><br>O64214; VG20_BPMD2 Gene 20 protein (99.18/5.20e-10)	head completion protein
9	5945	6301	+	prophage pi2 protein 37 [*Bacillus* phage 11143]<br><br>TPA: MAG TPA: putative tail component [*Siphoviridae* sp.]<br><br>TPA: MAG TPA: putative tail component [*Siphoviridae* sp.]	2.0e-46<br><br>3.0e-35<br><br>7.0e-32	-	-	COG5005; COG5005; Mu-like prophage protein gpG (99.62/3.20e-15)<br><br>Q01261; GPG_BPMU Putative capsid assembly protein G (98.86/1.40e-08)<br><br>Q04776; YG36_BPMV4 Uncharacterized protein ORF6 (98.55/2.30e-07)	putative tail completion protein

10	6298	6627	+	putative aminopeptidase [uncultured *Caudovirales* phage]<br><br>TPA: MAG TPA_asm: tail component [*Myoviridae* sp.]<br><br>TPA: MAG TPA: tail completion protein [*Myoviridae* sp.]	2.0e-62<br><br>7.0e-41<br><br>5.0e-40	-	-	O48448; COMPL_BPSPP Tail completion protein gp17 (98.63/9.70e-07)<br><br>O64216; VG22_BPMD2 Gene 22 protein (96.44/8.00e-02)	tail completion protein
11	6628	7221	+	putative major tail protein [uncultured *Caudovirales* phage]<br><br>major tail protein [*Bacillus* phage 11143]<br><br>putative major tail protein [*Exiguobacterium* phage vB_EauS-123]	4.0e-128<br><br>3.0e-127<br><br>2.0e-81	maj_tail_phi13 (5-192)	2.11e-39	O21879; TUBE_BPLSK Tail tube protein (100.0/1.10e-31)<br><br>Q05229; VG23_BPML5 Major tail protein Gp23 (97.25/1.70e-02)	tail tube protein
12	7228	7590	+	prophage pi2 protein 40 [*Bacillus* phage 11143]<br><br>TPA: MAG TPA: tail assembly chaperone protein [*Siphoviridae* sp.]<br><br>TPA: MAG TPA_asm: tail assembly chaperone protein [*Myoviridae* sp.]	5.0e-37<br><br>3.0e-36<br><br>8.0e-36	-	-	('-', '-')	tail assembly chaperone protein
13	7689	7805	+	TPA: MAG TPA: Ribosomal protein L14 [*Myoviridae* sp.]<br><br>TPA: MAG TPA: Protein of unknown function (DUF1108) [*Siphoviridae* sp.]	2.0e-03<br><br>2.0e-03	-	-	('-', '-')	hp
14	7821	11453	+	TMP repeat protein [Geo*Bacillus* phage GBSV1]<br><br>TMP repeat protein [*Bacillus* virus 1]	0.0e+00<br><br>0.0e+00	YqbO (1-696)<br><br>COG5412 (601-1071)	1.57e-48<br><br>8.00e-33	O21882; TMP_BPLSK Probable tape measure protein (100.0/2.40e-22)	tape measure protein

				putative TMP repeat protein [uncultured *Caudovirales* phage]	0.0e+00	SMC_prok_B (19-571)	1.38e-08	Q0PDK7; TMP_BPSPP Tail tape measure protein gp18 (99.97/2.00e-20)<br><br>E7DNB6; TMP_BPDP1 Tape measure protein (99.97/9.90e-20)	
15	11495	12952	+	tail family protein [*Bacillus* phage vB_BtS_BMBtp3]<br><br>tail family protein [*Bacillus* phage phi4J1]<br><br>tail family protein [Staphylococcus phage SpaA1]	0.0e+00<br><br>0.0e+00<br><br>0.0e+00	Sipho_tail (16-189)<br><br>phi3626_gp14_N (4-127)<br><br>YomH (4-181)	3.73e-34<br><br>4.02e-34<br><br>2.05e-20	COG4722; YomH; Phage-related protein (99.82/1.50e-18)<br><br>O48459; DIT_BPSPP Distal tail protein (99.8/6.70e-18)<br><br>O64221; VG27_BPMD2 Minor tail protein Gp27 (99.44/5.20e-12)	distal tail protein
16	12949	17331	+	putative minor structural protein [uncultured *Caudovirales* phage]<br><br>tail fiber domain-containing protein [*Bacillus* phage phi4B1]<br><br>minor structural protein [*Bacillus* phage phi4J1]	0.0e+00<br><br>0.0e+00<br><br>0.0e+00	put_anti_recept (29-351)<br><br>Prophage_tail (97-323)<br><br>Tar (355-593)	7.46e-70<br><br>3.06e-15<br><br>5.53e-08	Q0PDK6; FIBER_BPSPP Tail spike protein (99.96/5.90e-27)<br><br>COG4926; PblB; Phage-related protein (99.9/5.60e-22)<br><br>A8E283; VPN7_BPPHE Tail fiber protein (99.74/9.50e-16)	tail fiber protein
17	17347	17724	+	hp	hp	-	-	COG2002; AbrB; Bifunctional DNA-binding transcriptional regulator of stationary/sporulation/toxin gene expression and antitoxin c(99.19/7.80e-11) d2fy9a1; b.129.1.3 (A:1-54) Putative transition state	transcriptional regulator

								regulator ABH (98.69/2.20e-07)<br><br>d1yfba_; b.129.1.3 (A:) Transcription-state regulator AbrB, the N-terminal DNA recognition domain {*Bacillus* subtilis [TaxId: 1423(98.47/1.80e-06)	
18	17761	18186	+	holin family protein [*Bacillus* phage phi4J1]<br><br>holin [*Bacillus* phage BVE2]<br><br>holin [*Bacillus* phage vB_BtS_BMBtp13]	2.0e-87<br><br>1.0e-86<br><br>3.0e-86	Phage_holin_4_1<br>(22-127)	1.84e-37	COG4824; COG4824; Phage-related holin (Lysis protein) (99.96/9.10e-28)<br><br>Q9ZXD8; VLYS_BPPH1 Probable holin (99.96/6.60e-27)<br><br>P07539; HOLIN_BPPZA Antiholin (99.93/5.10e-24)	holin
19	18186	19118	+	N-acetylmuramoyl-L-alanine amidase [*Bacillus* phage Waukesha92]<br><br>N-acetylmuramoyl-L-alanine amidase [*Bacillus* phage vB_BthS-TP21T]<br><br>N-acetylmuramoyl-L-alanine amidase [*Bacillus* phage vB_BthS-HD29phi]	0.0e+00<br><br>0.0e+00<br><br>6.0e-128	CwlA<br>(3-167)<br><br>PGRP<br>(22-141)<br><br>sporang_Gsm<br>(186-307)	2.24e-56<br><br>2.38e-25<br><br>9.29e-09	d1yb0a1; d.118.1.1 (A:1-157) N-acetylmuramoyl-L-alanine amidase PlyG (99.85/2.70e-19)<br><br>COG5632; CwlA; N-acetylmuramoyl-L-alanine amidase CwlA [Cell wall/membrane/envelope biogenesis] (99.74/4.80e-16)<br><br>d2cb3a1; d.118.1.1 (A:174-344) Peptidoglycan-recognition protein-LE (99.63/3.00e-15)	N-acetylmuramoyl-L-alanine amidase

20	19384	20019	+	-	-	-	-	('-', '-')	hp
21	20044	20220	+	-	-	-	-	('-', '-')	hp
22	20213	20848	+	PIN domain protein [*CaldiBacillus* phage CBP1]<br><br>MULTISPECIES: type II toxin-antitoxin system VapC family toxin [*Bacillus* cereus group]	2.0e-06<br><br>5e-129	PIN_VapC-like (23-174)	2.03e-04	COG5573; COG5573; Predicted nucleic acid-binding protein, contains PIN domain [General function prediction only] (99.56/9.90e-13)<br><br>d1w8ia_; c.120.1.1 (A:) Hypothetical protein AF1683 (99.46/1.80e-12)<br><br>COG5378; COG5378; Predicted nucleic acid-binding protein, contains PIN domain [General function prediction only] (99.45/7.80e-12)	PIN domain-containing protein
23	21363	21040	-	hp	hp	-	-	Q9YJQ8; TIO_ATHV3 Protein tio (86.6/7.80e-01)	hp
24	21504	21722	+	hp	hp	-	-	COG2378; YafY; Predicted DNA-binding transcriptional regulator YafY, contains an HTH and WYL domains [Transcription] (99.11/4.50e-09)	transcriptional regulator
25	21747	22037	+	YolD-like family protein [*PaeniBacillus* phage Diva]<br><br>YolD-like protein [*PaeniBacillus* phage Xenia]	4.0e-06<br><br>4.0e-06	YolD (19-92)	2.36e-04	d1sg5a1; b.137.1.2 (A:1-86) Inhibitor of Rho Rof {Escherichia coli [TaxId: 562]} (95.69/2.30e-01)	YolD-like family protein

				YolD-like protein [*PaeniBacillus* phage Fern]	6.0e-06				
26	22884	22054	-	helix_turn_helix protein [*Bacillus* phage BM5]<br><br>putative cytosolic protein [*Bacillus* phage vB_BspM_MarvelLand]<br><br>DNA binding protein [*Bacillus* phage Spock]	2.0e-115<br><br>2.0e-111<br><br>2.0e-99	-	-	COG5529; COG5529; Pyocin large subunit [Secondary metabolites biosynthesis, transport and catabolism] (98.39/2.60e-06)<br><br>P19654; REPL_BPP1 Replication protein repL (98.02/3.70e-05)<br><br>P03688; VRPO_LAMBD Replication protein O (97.7/1.10e-04)	HTH domain-containing protein
27	24435	23272	-	site-specific integrase [*Bacillus* phage phi4B1]<br><br>integrase [*Bacillus* phage BMBtp1]<br><br>tyrosine recombinase XerC [Brevi*Bacillus* phage Emery]	0.0e+00<br><br>0.0e+00<br><br>2.0e-104	INT_ICEBs1_C_like (174-359)<br><br>XerC (55-379)<br><br>Phage_int_SAM_3 (63-117)	1.04e-46<br><br>1.93e-15<br><br>4.84e-11	P37317; VINT_BPSFV Integrase (100.0/2.20e-38)<br><br>P25426; VINT_BPMFR Integrase (100.0/5.30e-38)<br><br>P08320; VINT_BPP4 Integrase (100.0/5.10e-37)	site-specific integrase
28	24930	24505	-	repressor protein [*Bacillus* phage BMBtp1]<br><br>ImmA/IrrE family metallo-endopeptidase [*Bacillus* phage phi4B1]<br><br>ImmA/IrrE family metallo-endopeptidase [Listeria phage vB_LmoS_188]	5.0e-100<br><br>4.0e-93<br><br>7.0e-47	ImmA (8-141)	7.91e-25	P10426; YIM2_BPPH1 Uncharacterized immunity region protein 2 (99.92/1.50e-22)<br><br>COG2856; ImmA; Zn-dependent peptidase ImmA, M78 family [Posttranslational modification, protein	ImmA/IrrE family metallo-endopeptidase

								turnover, chaperones] (99.86/6.00e-20)<br><br>COG3800; COG3800; Predicted transcriptional regulator [General function prediction only] (99.8/2.50e-18)	
29	25368	24946	-	Cro/CI family transcriptional regulator [*Bacillus* phage BMBtp1]<br><br>DNA-binding helix-turn-helix protein [*Bacillus* phage phi4B1]<br><br>TPA: MAG TPA: repressor protein [*Siphoviridae* sp.]	8.0e-68<br><br>3.0e-66<br><br>4.0e-49	HTH_XRE (5-59)<br><br>HipB (2-88)	1.70e-08<br><br>6.22e-04	P06153; RPC_BPPH1 Immunity repressor protein (99.1/1.40e-07)<br><br>P04132; RPC_BPP2 Repressor protein C (98.46/3.80e-06)<br><br>COG5606; COG5606; Predicted DNA-binding protein, XRE-type HTH domain [General function prediction only] (98.23/1.80e-05)	transcriptional regulator (repressor protein)
30	25640	25825	+	transcriptional regulator [*Bacillus* phage phi4B1]<br><br>helix-turn-helix transcriptional regulator [Staphylococcus virus phiETA2]<br><br>helix-turn-helix transcriptional regulator [Staphylococcus virus 187]	8.0e-20<br><br>3.0e-10<br><br>4.0e-10	-	-	P04132; RPC_BPP2 Repressor protein C (98.9/5.70e-08)<br><br>d2icta_; a.35.1.3 (A:) Antitoxin HigA {Escherichia coli [TaxId: 562]} (98.32/8.40e-06)<br><br>COG5606; COG5606; Predicted DNA-binding protein, XRE-type HTH domain [General function	transcriptional regulator (repressor protein)

								prediction only] (98.14/2.70e-05)	
31	25825	26097	+	DUF771 domain-containing protein [*Bacillus* phage phi4B1]<br><br>TPA: MAG TPA: protein of unknown function (DUF771) [*Siphoviridae* sp.]<br><br>DUF771 domain-containing protein [Staphylococcus phage vB_SauS-phiIPLA88]	4.0e-53<br><br>5.0e-06<br><br>4.0e-03	DUF771 (32-90)	2.16e-10	COG4707; COG4707; Prophage pi2 protein 07 [Mobilome: prophages, transposons] (99.77/1.10e-18)<br><br>P25135; VG090_BPPF1 10.1 kDa protein OS=Pseudomonas phage Pf1 OX=2011081 PE=4 SV=1(98.63/5.10e-08)<br><br>d1j9ia_; a.6.1.5 (A:) Terminase gpNU1 subunit domain {Bacteriophage lambda [TaxId: 10710]} (98.61/1.10e-07)	DUF771 domain-containing protein
32	26112	26267	+	putative NHN endonuclease [*Bacillus* phage vB_*Bacillus*_1020A]<br><br>gp68 [Listeria phage B054]<br><br>TPA: MAG TPA: Metallo-beta-lactamase superfamily [*Myoviridae* sp.]	6.0e-13<br><br>1.0e-07<br><br>2.0e-06	-	-	Q914G0; Y072_SIFVH Uncharacterized protein 72 (68.48/4.10e+00)	hp
33	26284	27102	+	ORF6C domain-containing protein [*Bacillus* phage phi4J1]<br><br>antirepressor [*Bacillus* phage BMBtp1]<br><br>antirepressor-like protein [*Bacillus* thuringiensis phage MZTP02]	0.0e+00<br><br>2.0e-174<br><br>6.0e-149	ORF6C (147-259)	2.19e-31	P03037; RANT_BPP22 Antirepressor protein ant (98.17/5.00e-05)<br><br>COG3646; pRha; Phage regulatory protein Rha [Mobilome: prophages, transposons] (97.36/3.80e-04)	transcriptional regulator (antirepressor protein)

								P19655; ANT_BPP1 Antirepressor protein 1 (97.08/7.10e-03)	
34	27114	27302	+	hp	hp	-	-	COG4803; COG4803; Uncharacterized membrane protein [Function unknown] (76.57/7.70e+00)	hp
35	27329	27763	+	replication terminator protein [Aeri*Bacillus* phage AP45]<br><br>replication terminator protein [*PaeniBacillus* phage Vegas]<br><br>replication terminator protein [*PaeniBacillus* phage Dragolir]	6.0e-39<br><br>7.0e-28<br><br>1.0e-27	-	-	('-', '-')	replication terminator protein
36	27782	28495	+	hp	hp	-	-	COG5532; yfdQ; Uncharacterized conserved protein YfdQ, DUF2303 family [Function unknown].(94.21/5.40e+00)	hp
37*	28495	28710	+	hp	hp	-	-	cd14652; Seven_helix_coil Seven_helix_coil. other structures are fusion proteins (84.14/9.80e-01)	hp
38	29077	30012	+	putative replication protein [uncultured *Caudovirales* phage]<br><br>replication initiation protein [*Bacillus* phage BMBtp1]<br><br>DnaD domain protein [*Bacillus* phage phi4J1]	0.0e+00<br><br>0.0e+00<br><br>1.0e-162	DnaD (144-287)<br><br>DnaD_dom (184-245)	7.70e-15<br><br>4.17e-13	COG3935; DnaD; DNA replication protein DnaD [Replication, recombination and repair] (99.27/1.30e-10)<br><br>P03688; VRPO_LAMBD Replication protein (98.21/2.00e-06)	DNA replication protein DnaD

								COG3611; DnaB; Replication initiation and membrane attachment protein DnaB [Replication, recombination and repair] (98.5/4.90e-06)	
39	30024	30503	+	putative structural protein [Vibrio phage vB_VpP_DE10]	3.0e-04	-	-	P15854; GP166_BPPH5 Gene product 16.6 (94.63/5.80e-03)<br><br>COG1675; TFA1; Transcription initiation factor IIE, alpha subunit [Transcription] (94.83/2.10e-02)<br><br>d1zina2; g.41.2.1 (A:126-160) Microbial and mitochondrial ADK, insert "zinc finger" domain {*Bacillus* stearothermophilus [TaxId: 1(91.79/4.60e-02)	hp
40	30496	30726	+	hp	hp	-	-	('-', '-')	hp
41	30750	31307	+	lysozyme-like protein [*Bacillus* phage vB_BtS_BMBtp14]<br><br>gp38 [*Bacillus* phage TP21-L]<br><br>TPA: MAG TPA: PVL ORF 50 like protein [*Myoviridae* sp.]	1.0e-84<br><br>1.0e-77<br><br>4.0e-19	-	-	d1rh6a_; a.6.1.7 (A:) Excisionase Xis (89.7/5.10e-01)	hp
42	31346	31780	+	hp	hp	-	-	Q02406; Y12J_BPT4 Uncharacterized 7.3 kDa protein in Gp30-rIII intergenic region (93.52/2.40e-01)	hp

43	31905	32441	+	putative dUTPase [uncultured *Caudovirales* phage]<br><br>dUTP diphosphatase [*Bacillus* phage vB_BtS_BMBtp3]<br><br>dUTPase [*Bacillus* phage vB_BthS-TP21T]	1.0e-117<br><br>7.0e-24<br><br>1.0e-22	dUTPase_2 (7-178)<br><br>NTP-PPase_dUTPase (11-93)<br><br>Dut2 (6-178)	2.91e-23<br><br>1.67e-18<br><br>5.30e-13	COG4508; Dut2; Dimeric dUTPase, all-alpha-NTP-PPase (MazG) superfamily [Nucleotide transport and metabolism].(100.0/2.20e-32)<br><br>d1w2ya_; a.204.1.1 (A:) Type II deoxyuridine triphosphatase (99.96/1.40e-28)<br><br>d1ogla_; a.204.1.1 (A:) Type II deoxyuridine triphosphatase (99.9/1.30e-22)	dUTPase
44	32461	32601	+	hp	hp	-	-	KOG3637; Vitronectin receptor, alpha subunit [Extracellular structures] (83.84/1.50e+00)	hp
45	32603	33394	+	HflC/HflK family inner membrane protein [*Bacillus* phage Spock]<br><br>band 7 protein [*Bacillus* phage B4]<br><br>membrane protein [*Bacillus* phage BigBertha]	8.0e-161<br><br>1.0e-158<br><br>5.0e-152	SPFH_prohibitin (27-225)<br><br>HflC (1-230)<br><br>PHB (25-191)	6.62e-47<br><br>1.62e-09<br><br>6.24e-05	KOG3083; Prohibitin [Posttranslational modification, protein turnover, chaperones] (100.0/6.90e-35)<br><br>KOG3090; Prohibitin-like protein [Posttranslational modification, protein turnover, chaperones] (100.0/4.50e-34)<br><br>KOG2621; Prohibitins and stomatins of the PID superfamily [Energy	SPFH domain-containing membrane protein (Prediction: Signal peptide (Sec/SPI)<br><br>Cleavage site between pos. 35 and 36: GHA-GV. Probability: 0.3497)

								production and conversion] (100.0/7.40e-30)	
46	33419	34006	+	putative recombination protein U [uncultured *Caudovirales* phage]<br><br>Holliday junction resolvase RecU [Staphylococcus phage SpaA1]<br><br>putative recombination protein U [*Bacillus* phage vB_BtS_BMBtp3]	2.0e-122<br><br>2.0e-85<br><br>2.0e-84	RecU (28-188)	4.66e-59	COG3331; PrfA; Penicillin-binding protein-related factor A, putative recombinase (100.0/9.00e-34)<br><br>d1rzna_; c.52.1.28 (A:) Recombination protein U (RecU)/PBP related factor A (PrfA) (100.0/4.10e-32)<br><br>d1ob8a_; c.52.1.18 (A:) Holliday-junction resolvase SSO1176 (99.19/6.70e-10)	Holliday junction resolvase RecU
47	34003	34326	+	Zn-finger protein fused to HTH domain [Staphylococcus phage SpaA1]	2.0e-22	-	-	d1ijwc_; a.4.1.2 (C:) HIN recombinase (DNA-binding domain) (98.11/1.90e-05)<br><br>d1gdta1; a.4.1.2 (A:141-183) gamma,delta resolvase (C-terminal domain) (97.86/1.10e-04)<br><br>d1rr7a_; a.4.1.14 (A:) Middle operon regulator, Mor (97.91/1.90e-04)	hp
48	34458	34967	+	putative sigma-70 family RNA polymerase sigma factor [uncultured *Caudovirales* phage]<br><br>sigma-70 family RNA polymerase sigma factor [*Bacillus* phage Waukesha92]	3.0e-94<br><br>2.0e-53	PRK06930 (1-168)<br><br>sigma70-ECF (77-161)<br><br>Sigma70_r4	6.59e-61<br><br>2.48e-09<br><br>5.48e-09	d1or7a1; a.4.13.2 (A:120-187) SigmaE factor (RpoE) (98.44/5.80e-06)<br><br>P06227; RP34_BPSP1 RNA polymerase sigma GP34 factor (98.33/7.10e-06)	sigma-70 family RNA polymerase sigma factor

				sigma-70 family RNA polymerase sigma factor [*Bacillus* phage vB_BtS_BMBtp3]	5.0e-53	(104-157)		d1ttya_; a.4.13.2 (A:) Sigma70 (SigA, RpoD) (98.29/9.20e-06)	
49	35421	35630	+	hp	hp	-	-	('-', '-')	hp
50	35696	35875	+	hp	hp	-	-	Q9J555; A13_FOWPN Virion membrane protein A13 homolog (80.17/6.70e+00)	hp
51	35877	36089	+	DNA-binding protein [*Bacillus* phage Deep-Purple]<br><br>helix turn helix domain protein [*CaldiBacillus* phage CBP1]<br><br>DNA-protecting protein DprA [Thermus phage phi OH2]	2.0e-23<br><br>5.0e-10<br><br>3.0e-06	HTH_17(20-66)	1.59e-04	d1j9ia_; a.6.1.5 (A:) Terminase gpNU1 subunit domain (99.04/6.90e-09)<br><br>P25135; VG090_BPPF1 10.1 kDa protein (98.96/1.40e-08)<br><br>P51705; VCOX_BPHC1 Regulatory protein cox (98.86/2.30e-08)	HTH domain-containing protein
52	36147	36449	+	TPA: MAG TPA: HNH endonuclease [*Siphoviridae* sp.]<br><br>TPA: MAG TPA: HNH endonuclease bacteriophage, HNH Endonuclease, DNA.52A [*Siphoviridae* sp.]<br><br>TPA: MAG TPA: HNH endonuclease bacteriophage, HNH Endonuclease, DNA.52A [*Siphoviridae* sp.]	2.0e-37<br><br>4.0e-32<br><br>5.0e-32	-	-	d4ogca2; d.4.1.8 (A:513-673) CRISPR-associated endonuclease Cas9/Csn1, HNH domain (97.74/9.40e-05)<br><br>d5axwa2; d.4.1.8 (A:484-630) CRISPR-associated endonuclease Cas9/Csn1, HNH domain (97.19/1.50e-03)<br><br>d4oo8a2; d.4.1.8 (A:775-907) CRISPR-associated endonuclease Cas9/Csn1,	HNH endonuclease

								HNH domain (97.08/1.80e-03)	
53	36452	36847	+	hp	hp	-	-	('-', '-')	hp

* – CDS37 (locus_tag: phageB13_37; protein_id UUW40223; location 28495-28710), predicted by RAST, encodes a hypothetical protein. There is an alternative overlapping CDS which was discovered manually and is located downstream (28463-28981) of the RAST-predicted CDS37. The alternative CDS has an RBS that is slightly closer to the proposed start codon compared with the RBS of the RAST-predicted CDS37. Since both the RAST-predicted and alternative CDSs encode hypothetical proteins, it is very difficult to tell which one is the real gene.

Table S3. The large terminase subunit proteins of B13 and phages with well-studied DNA packaging mechanisms used for phylogenetic inference.

Terminus type		Phage	Number GenBank (terminase large subunit)	Source
**Exact direct terminal repeats**	Short direct terminal repeats (T7)	*Pseudomonas* phage Pf-10	YP_009145642.1	[3]
		Enterobacteria phage T7	QRE00040.1	[4]
		Enterobacteria phage T3	YP_009792972.1	[5]
		Yersinia phage phiYeO3-12	NP_052122.1	[6]
		Pseudomonas phage phi15	YP_004286227.1	[7]
		Pseudomonas phage PFP1	YP_009804025.1	[8]
	Short direct terminal repeats (N4)	*Escherichia phage N4*	YP_950546.1	[9]
		Achromobacter phage JWDelta	AHC56597.1	[10]
		Erwinia phage vB_EamP-S6	YP_007005834.1	[11]
	Short direct terminal repeats (c-st)	*Clostridium* phage c-st	YP_398598.1	[12]
		Bacillus phage Izhevsk	QIW89903.1	[13]
		Bacillus phage vB_BanS-Tsamsa	AGI11737.1	[14]
		Bacillus phage Basilisk	AGR46580.1	[15]
	Long DTRs (SPO1)	*Bacillus* virus SPO1	YP_002300330.1	[16]
		Listeria phage A511	YP_001468454.1	[17]
		Brochothrix phage A9	YP_004301396.1	[18]
	Long DTRs (T5)	*Enterobacteria* phage T5	YP_006983.1	[4]
		Salmonella virus SPC35	YP_004306624.1	[19]
		Providencia phage vB_PreS_PR1	YP_009599184.1	[20]
**Cohesive ends**	5′cos ends (lambda)	Bacteriophage N15	NP_046897.1	[21]
		Enterobacteria phage lambda	NP_040581.1	[4]
	5′cos ends (P2)	*Escherichia* virus P2	NP_046758.1	[4,22]
		Escherichia virus 186	NP_052251.1	[23]
		Pseudomonas phage phiCTX	NP_490600.1	[24]
	3′cos ends (HK97)	*Escherichia* virus HK97	NP_037698.1	[25]
		Escherichia virus HK022	NP_037663.1	[25]
		RHizobium phage 16-3	YP_002117560.1	[26]

		***Bacillus* phage B13**	UUW40188.1	-
**Host DNA at termini**	Host ends (Mu)	*Escherichia* virus Mu	AAF01106.1	[27]
		Pseudomonas phage B3	YP_164067.1	[28]
		Burkholderia virus BcepMu	YP_024701.1	[29]
	Host ends (D3112)	*Pseudomonas* virus MP22	YP_001469154.1	[30]
		Haemophilus phage SuMu	YP_007002934.1	[31]
		Pseudomonas virus D3112	NP_938233.1	[30]
**Circularly permuted direct terminal repeats**	Headful (P22)	*Salmonella* virus P22	YP_063734.1	[32]
		Salmonella phage ST64T	NP_720326.1	[33]
		Enterobacteria phage LP7	AAA88220.1	[34]
	Headful (Sf6)	*Shigella* phage Sf6	NP_958178.1	[35]
		Hamiltonella virus APSE1	NP_050979.1	[36]
		Enterobacteria phage CUS-3	ABQ88401.1	[37]
	Headful (933W)	*Enterobacteria* phage 933W	NP_049511.1	[38]
		Burkholderia virus Bcep22	NP_944278.1	[39]
	Headful (phiKZ)	*Pseudomonas* phage phiKZ	NP_803591.1	[40]
		Pseudomonas phage 201phi2-1	YP_001956731.1	[41]
		Erwinia phage phiEaH2	YP_007237828.1	[42]
	Headful (T4)	*Enterobacteria* phage T4	NP_049776.1	[43]
		Vibrio phage KVP40	NP_899601.1	[43]
		Enterobacteria phage RB49	NP_891724.1	[44]
	Headful (phiPLPE)	*Yersinia* phage PY100	CAJ28416.1	[45]
		Klebsiella phage JD001	YP_007392855.1	[46]
		Iodobacterteriophage phiPLPE	YP_002128452.1	[46]
	Headful (SPP1)	*Bacillus* phage vB_BcM_Sam46	QIQ61203.1	[47]
		Bacillus phage SPP1	NP_690654.1	[48]
		Staphylococcus virus CNPH82	YP_950600.1	[49]
	Headful (B83)	*Bacillus* Phage vB_BtS_B83	QCQ57785.1	[50]
		Bacillus phage vB_BtS_BMBtp14	YP_009830709.1	[50]
**Covalent terminal protein (phi29)**	Protein-primed mechanism [Longás,2008]	*Bacillus* phage phi29	YP_002004545.1	[51–53]
		Bacillus phage Nf	YP_009910733.1	[53]

Table S4. Phage genomes used for phylogenetic inference.

№	Name	Genome Accession number	Genome length	GC-content, %	SDCs	Number of tRNAs/tmRNAs	BLASTn nucleotide identity to B13, %*	Proteins shared with B13**	
								number	%
1	*Bacillus* phage B13	OP066531	36,864	34.8	53	0/0	-	-	-
2	*Bacillus* phage BMBtp1	KT852578.1	35,838	34.9	58	0/0	27.73	26	46.85
3	Uncultured *Caudovirales* phage clone 9AX_2, partial genome	MF417893.1	43,032	34.7	61	0/0	29.20	22	38.60
4	*Bacillus* phage phi4J1	NC_029008.1	41,486	35.9	64	0/0	31.28	22	37.60
5	*Bacillus* phage vB_BanS_Athena	OK500002.1	37,369	35.3	62	0/0	21.54	16	27.83
6	*Bacillus* phage Waukesha92	NC_025424.1	45,648	35.5	71	0/0	12.60	13	20.97
7	*Bacillus* phage phiS58	KT970646.1	46,635	35.4	70	0/0	12.60	13	21.14
8	*Bacillus* phage vB_BthS-TP21T	MK843319.1	51,949	35.5	82	0/0	12.60	13	19.26
9	*Bacillus* phage vB_BtS_BMBtp3	NC_028748.2	51,366	35.4	72	0/0	9.12	10	16.00
10	*Bacillus anthracis* phage Cherry	DQ222851.1	36,615	35.3	53	0/0	8.48	9	17.00
11	*Bacillus* phage Gamma	NC_007458.1	37,253	35.2	55	0/0	8.49	9	16.70
12	*Bacillus anthracis* phage Gamma isolate d'Herelle	DQ289556.1	37,373	35.1	56	0/0	7.85	9	16.50
13	*Bacillus* phage AP631	MK085976.1	39,549	35.0	56	0/0	7.78	9	16.50
14	*Bacillus* phage WBeta	NC_007734.1	40,867	35.3	57	0/0	7.84	9	16.36

15	*Bacillus* phage phi4B1	NC_028886.1	38,663	35.9	64	0/0	15.31	9	15.38
16	*Bacillus* phage Fah	NC_007814.1	37,974	34.9	54	0/0	7.79	8	14.95
17	*Bacillus* phage phIS3501	NC_019502.1	44,401	34.9	69	1/0	11.15	7	11.48
18	*Bacillus* phage phiCM3	NC_023599.1	38,772	35.5	56	0/0	9.60	6	11.01
19	*Bacillus* phage BtCS33	NC_018085.1	41,992	35.2	59	0/0	7.57	6	10.71
20	*Bacillus* phage BceA1	NC_048628.1	42,932	35.7	63	0/0	9.10	6	10.35
21	*Staphylococcus* phage SpaA1	NC_018277.1	42,784	35.6	65	0/0	9.10	6	10.17
22	*Bacillus* phage PfEFR-5	NC_031055.1	43,773	35.	68	0/0	7.81	6	9.92
23	*Bacillus* phage PfEFR-4	NC_048641.1	43,223	35.4	69	0/0	7.81	6	9.84
24	*Bacillus* phage vB_BceS-MY192	NC_048633.1	44,696	35.0	66	0/0	7.10	5	8.40
25	*Bacillus* phage vB_BtS_B83	NC_048762.1	49,952	35.8	71	0/0	8.49	4	6.45
26	*Bacillus* phage vB_BtS_BMBtp14	NC_048640.1	50,740	36.8	77	0/0	3.41	4	6.15
27	*Bacillus* phage TP21-L	NC_011645.1	37,456	37.	61	0/0	2.97	3	5.26
28	*Bacillus* phage vB_BtS_BMBtp2	NC_019912.1	36,932	37.8	55	0/0	2.72	2	
29	*Bacillus* phage phBC6A51	NC_004820.1	61,395	37.7	94	0/0	5.56	0	
30	*Bacillus* phage vB_BboS-125	NC_048735.1	58,528	48.6	87	0/0	0.00	0	

*Determined using BLASTn compared to B13 (multiplying % coverage by % identity); **Determined using GET_HOMOLOGUES (COGtriangles algorithm, -G -t 0 -C 75).
The additional six phage genomes found by the BLASTn search using the whole B13 genome sequence as the query are light gray.

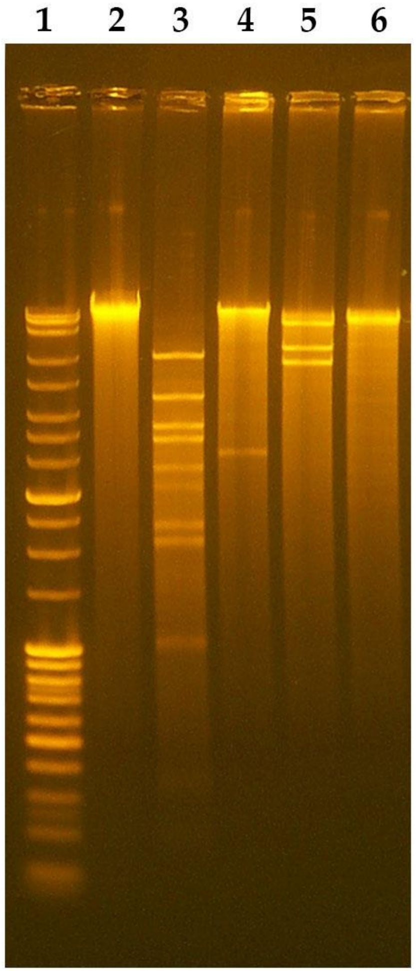

Figure S1. Restriction analysis of B13 genomic DNA. This is the original gel image used to generate Figure 6, A in the main text. Kodak EDAS 290 Gel Documentation System ("Kodak") was used to capture the image. 1 – molecular weight markers; 2 – intact phage DNA; 3 – HindIII; 4 – BamHI; 5 – BglII; 6 – PstI.

References

1. Hong, H.A.; Le, H.D.; Cutting, S.M. The Use of Bacterial Spore Formers as Probiotics. *FEMS Microbiol Rev* 2005, *29*.

2. Sorokin, A.; Candelon, B.; Guilloux, K.; Galleron, N.; Wackerow-Kouzova, N.; Ehrlich, S.D.; Bourguet, D.; Sanchis, V. Multiple-Locus Sequence Typing Analysis of Bacillus Cereus and Bacillus Thuringiensis Reveals Separate Clustering and a Distinct Population Structure of Psychrotrophic Strains. *Appl Environ Microbiol* **2006**, *72*, doi:10.1128/AEM.72.2.1569-1578.2006.

3. Kazantseva, O.A.; Buzikov, R.M.; Pilipchuk, T.A.; Valentovich, L.N.; Kazantsev, A.N.; Kalamiyets, E.I.; Shadrin, A.M. The Bacteriophage Pf-10—A Component of the Biopesticide "Multiphage" Used to Control Agricultural Crop Diseases Caused by Pseudomonas Syringae. *Viruses* **2022**, *14*, doi:10.3390/v14010042.

4. Casjens, S.R.; Gilcrease, E.B. Determining DNA Packaging Strategy by Analysis of the Termini of the Chromosomes in Tailed-Bacteriophage Virions. *Methods Mol Biol* **2009**, *502*, doi:10.1007/978-1-60327-565-1_7.

5. Pajunen, M.I.; Elizondo, M.R.; Skurnik, M.; Kieleczawa, J.; Molineux, I.J. Complete Nucleotide Sequence and Likely Recombinatorial Origin of Bacteriophage T3. *J Mol Biol* **2002**, *319*, doi:10.1016/S0022-2836(02)00384-4.

6. Pajunen, M.; Kiljunen, S.; Skurnik, M. Bacteriophage ΦYeO3-12, Specific for Yersinia Enterocolitica Serotype O:3, Is Related to Coliphages T3 and T7. *J Bacteriol* **2000**, *182*, doi:10.1128/JB.182.18.5114-5120.2000.

7. Cornelissen, A.; Ceyssens, P.J.; T'Syen, J.; van Praet, H.; Noben, J.P.; Shaburova, O. v.; Krylov, V.N.; Volckaert, G.; Lavigne, R. The T7-Related Pseudomonas Putida Phage Φ15 Displays Virion-Associated Biofilm Degradation Properties. *PLoS One* **2011**, *6*, doi:10.1371/journal.pone.0018597.

8. Li, M.; Chen, X.; Ma, Y.; Li, Z.; Zhao, Q. Complete Genome Sequence of PFP1, a Novel T7-like Pseudomonas Fluorescens Bacteriophage. *Arch Virol* **2018**, *163*, doi:10.1007/s00705-018-3979-3.

9. Wittmann, J.; Turner, D.; Millard, A.D.; Mahadevan, P.; Kropinski, A.M.; Adriaenssens, E.M. From Orphan Phage to a Proposed New Family–the Diversity of N4-like Viruses. *Antibiotics* **2020**, *9*, doi:10.3390/antibiotics9100663.

10. Ma, Y.; Li, E.; Qi, Z.; Li, H.; Wei, X.; Lin, W.; Zhao, R.; Jiang, A.; Yang, H.; Yin, Z.; et al. Isolation and Molecular Characterisation of Achromobacter Phage PhiAxp-3, an N4-like Bacteriophage. *Sci Rep* **2016**, *6*, doi:10.1038/srep24776.

11. Born, Y.; Fieseler, L.; Marazzi, J.; Lurz, R.; Duffy, B.; Loessner, M.J. Novel Virulent and Broad-Host-Range Erwinia Amylovora Bacteriophages Reveal a High Degree of Mosaicism and a Relationship to Enterobacteriaceae Phages. *Appl Environ Microbiol* **2011**, *77*, doi:10.1128/AEM.03022-10.

12. Sakaguchi, Y.; Hayashi, T.; Kurokawa, K.; Nakayama, K.; Oshima, K.; Fujinaga, Y.; Ohnishi, M.; Ohtsubo, E.; Hattori, M.; Oguma, K. The Genome Sequence of Clostridium Botulinum Type C Neurotoxin-Converting Phage and the Molecular Mechanisms of Unstable Lysogeny. *Proc Natl Acad Sci U S A* **2005**, *102*, doi:10.1073/pnas.0505503102.

13. Skorynina, A. v.; Piligrimova, E.G.; Kazantseva, O.A.; Kulyabin, V.A.; Baicher, S.D.; Ryabova, N.A.; Shadrin, A.M. Bacillus-Infecting Bacteriophage Izhevsk Harbors Thermostable Endolysin with Broad Range Specificity. *PLoS One* **2020**, *15*, doi:10.1371/journal.pone.0242657.

14. Grose, J.H.; Jensen, J.D.; Merrill, B.D.; Fisher, J.N.B.; Burnett, S.H.; Breakwell, D.P. Genome Sequences of Three Novel Bacillus Cereus Bacteriophages. *Genome Announc* **2014**, *2*, doi:10.1128/genomea.01118-13.

15. Stewart, C.R.; Casjens, S.R.; Cresawn, S.G.; Houtz, J.M.; Smith, A.L.; Ford, M.E.; Peebles, C.L.; Hatfull, G.F.; Hendrix, R.W.; Huang, W.M.; et al. The Genome of Bacillus Subtilis Bacteriophage SPO1. *J Mol Biol* **2009**, *388*, doi:10.1016/j.jmb.2009.03.009.

16. Klumpp, J.; Dorscht, J.; Lurz, R.; Bielmann, R.; Wieland, M.; Zimmer, M.; Calendar, R.; Loessner, M.J. The Terminally Redundant, Nonpermuted Genome of Listeria Bacteriophage A511: A Model for the SPO1-like Myoviruses of Gram-Positive Bacteria. *J Bacteriol* **2008**, *190*, doi:10.1128/JB.00461-08.

17. Kilcher, S.; Loessner, M.J.; Klumpp, J. Brochothrix Thermosphacta Bacteriophages Feature Heterogeneous and Highly Mosaic Genomes and Utilize Unique Prophage Insertion Sites. *J Bacteriol* **2010**, *192*, doi:10.1128/JB.00709-10.

18. Kim, M.; Ryu, S. Characterization of a T5-like Coliphage, SPC35, and Differential Development of Resistance to SPC35 in Salmonella Enterica Serovar Typhimurium and Escherichia Coli. *Appl Environ Microbiol* **2011**, *77*, doi:10.1128/AEM.02504-10.

19. Oliveira, H.; Pinto, G.; Hendrix, H.; Noben, J.-P.; Gawor, J.; Kropinski, A.M.; Łobocka, M.; Lavigne, R.; Azeredo, J. A Lytic Providencia Rettgeri Virus of Potential Therapeutic Value Is a Deep-Branching Member of the T5virus Genus. *Appl Environ Microbiol* **2017**, *83*, doi:10.1128/AEM.01567-17.

20. Ravin, N. v. N15: The Linear Phage-Plasmid. *Plasmid* 2011, *65*.

21. Christie, G.E.; Calendar, R. P2 Bacteriophage. *Bacteriophage* **2016**, *6*, doi:10.1080/21597081.2016.1145782.

22. Bullas, L.R.; Mostaghimi, A.R.; Arensdorf, J.J.; Rajadas, P.T.; Zuccarelli, A.J. Salmonella Phage PSP3, Another Member of the P2-like Phage Group. *Virology* **1991**, *185*, doi:10.1016/0042-6822(91)90573-T.

23. Nakayama, K.; Kanaya, S.; Ohnishi, M.; Terawaki, Y.; Hayashi, T. The Complete Nucleotide Sequence of PhiCTX, a Cytotoxin-Converting Phage of Pseudomonas Aeruginosa: Implications for Phage Evolution and Horizontal Gene Transfer via Bacteriophages. *Mol Microbiol* **1999**, *31*, 399–419, doi:10.1046/j.1365-2958.1999.01158.x.

24. Juhala, R.J.; Ford, M.E.; Duda, R.L.; Youlton, A.; Hatfull, G.F.; Hendrix, R.W. Genomic Sequences of Bacteriophages HK97 and HK022: Pervasive Genetic Mosaicism in the Lambdoid Bacteriophages. *J Mol Biol* **2000**, *299*, doi:10.1006/jmbi.2000.3729.

25. Ganyu, A.; Csiszovszki, Z.; Ponyi, T.; Kern, A.; Buzás, Z.; Orosz, L.; Papp, P.P. Identification of Cohesive Ends and Genes Encoding the Terminase of Phage 16-3. *J Bacteriol* **2005**, *187*, doi:10.1128/JB.187.7.2526-2531.2005.

26. Morgan, G.J.; Hatfull, G.F.; Casjens, S.; Hendrix, R.W. Bacteriophage Mu Genome Sequence: Analysis and Comparison with Mu-like Prophages in Haemophilus, Neisseria and Deinococcus. *J Mol Biol* **2002**, *317*, doi:10.1006/jmbi.2002.5437.

27. Braid, M.D.; Silhavy, J.L.; Kitts, C.L.; Cano, R.J.; Howe, M.M. Complete Genomic Sequence of Bacteriophage B3, a Mu-like Phage of Pseudomonas Aeruginosa. *J Bacteriol* **2004**, *186*, doi:10.1128/JB.186.19.6560-6574.2004.

28. Summer, E.J.; Gonzalez, C.F.; Carlisle, T.; Mebane, L.M.; Cass, A.M.; Savva, C.G.; LiPuma, J.J.; Young, R. Burkholderia Cenocepacia Phage BcepMu and a Family of Mu-like Phages Encoding Potential Pathogenesis Factors. *J Mol Biol* **2004**, *340*, doi:10.1016/j.jmb.2004.04.053.

29. Heo, Y.J.; Chung, I.Y.; Choi, K.B.; Lau, G.W.; Cho, Y.H. Genome Sequence Comparison and Superinfection between Two Related Pseudomonas Aeruginosa Phages, D3112 and MP22. *Microbiology (N Y)* **2007**, *153*, doi:10.1099/mic.0.2007/007260-0.

30. Zehr, E.S.; Tabatabai, L.B.; Bayles, D.O. Genomic and Proteomic Characterization of SuMu, a Mu-like Bacteriophage Infecting Haemophilus Parasuis. *BMC Genomics* **2012**, *13*, doi:10.1186/1471-2164-13-331.

31. Byl, C. v.; Kropinski, A.M. Sequence of the Genome of Salmonella Bacteriophage P22. *J Bacteriol* **2000**, *182*, doi:10.1128/JB.182.22.6472-6481.2000.

32. Mmolawa, P.T.; Schmieger, H.; Tucker, C.P.; Heuzenroeder, M.W. Genomic Structure of the Salmonella Enterica Serovar Typhimurium DT 64 Bacteriophage ST64T: Evidence for Modular Genetic Architecture. *J Bacteriol* **2003**, *185*, doi:10.1128/JB.185.11.3473-3475.2003.

33. Bernhard Petri, J.; Schmieger, H. Isolation of Fragments with Pac Function for Phage P22 from Phage LP7 DNA and Comparison of Packaging Gene 3 Sequences. *Gene* **1990**, *88*, doi:10.1016/0378-1119(90)90058-Y.

34. Zhao, H.; Christensen, T.E.; Kamau, Y.N.; Tang, L. Structures of the Phage Sf6 Large Terminase Provide New Insights into DNA Translocation and Cleavage. *Proc Natl Acad Sci U S A* **2013**, *110*, doi:10.1073/pnas.1301133110.

35. van der Wilk, F.; Dullemans, A.M.; Verbeek, M.; van den Heuvel, J.F.J.M. Isolation and Characterization of APSE-1, a Bacteriophage Infecting the Secondary Endosymbiont of Acyrthosiphon Pisum. *Virology* **1999**, *262*, doi:10.1006/viro.1999.9902.

36. King, M.R.; Vimr, R.P.; Steenbergen, S.M.; Spanjaard, L.; Plunkett, G.; Blattner, F.R.; Vimr, E.R. Escherichia Coli K1-Specific Bacteriophage CUS-3 Distribution and Function in Phase-Variable Capsular Polysialic Acid O Acetylation. *J Bacteriol* **2007**, *189*, doi:10.1128/JB.00657-07.

37. Plunkett, G.; Rose, D.J.; Durfee, T.J.; Blattner, F.R. Sequence of Shiga Toxin 2 Phage 933W from Escherichia Coli O157:H7: Shiga Toxin as a Phage Late-Gene Product? *J Bacteriol* **1999**, *181*, doi:10.1128/jb.181.6.1767-1778.1999.

38. Gill, J.J.; Summer, E.J.; Russell, W.K.; Cologna, S.M.; Carlile, T.M.; Fuller, A.C.; Kitsopoulos, K.; Mebane, L.M.; Parkinson, B.N.; Sullivan, D.; et al. Genomes and Characterization of Phages Bcep22 and BcepIL02, Founders of a Novel Phage Type in Burkholderia Cenocepacia. *J Bacteriol* **2011**, *193*, doi:10.1128/JB.05287-11.

39. Lecoutere, E.; Ceyssens, P.J.; Miroshnikov, K.A.; Mesyanzhinov, V. v.; Krylov, V.N.; Noben, J.P.; Robben, J.; Hertveldt, K.; Volckaert, G.; Lavigne, R. Identification and Comparative Analysis of the Structural Proteomes of ΦKZ and EL, Two Giant Pseudomonas Aeruginosa Bacteriophages. *Proteomics* **2009**, *9*, doi:10.1002/pmic.200800727.

40. Thomas, J.A.; Rolando, M.R.; Carroll, C.A.; Shen, P.S.; Belnap, D.M.; Weintraub, S.T.; Serwer, P.; Hardies, S.C. Characterization of Pseudomonas Chlororaphis Myovirus 201φ{symbol}2-1 via Genomic Sequencing, Mass Spectrometry, and Electron Microscopy. *Virology* **2008**, *376*, doi:10.1016/j.virol.2008.04.004.

41. Dömötör, D.; Becságh, P.; Rákhely, G.; Schneider, G.; Kovács, T. Complete Genomic Sequence of Erwinia Amylovora Phage PhiEaH2. *J Virol* **2012**, *86*, doi:10.1128/jvi.01870-12.

42. Miller, E.S.; Heidelberg, J.F.; Eisen, J.A.; Nelson, W.C.; Durkin, A.S.; Ciecko, A.; Feldblyum, T. v.; White, O.; Paulsen, I.T.; Nierman, W.C.; et al. Complete Genome Sequence of the Broad-Host-Range Vibriophage KVP40: Comparative Genomics of a T4-Related Bacteriophage. *J Bacteriol* **2003**, *185*, doi:10.1128/JB.185.17.5220-5233.2003.

43. Desplats, C.; Dez, C.; Tétart, F.; Eleaume, H.; Krisch, H.M. Snapshot of the Genome of the Pseudo-T-Even Bacteriophage RB49. *J Bacteriol* **2002**, *184*, doi:10.1128/JB.184.10.2789-2804.2002.

44. Schwudke, D.; Ergin, A.; Michael, K.; Volkmar, S.; Appel, B.; Knabner, D.; Konietzny, A.; Strauch, E. Broad-Host-Range Yersinia Phage PY100: Genome Sequence, Proteome Analysis of Virions, and DNA Packaging Strategy. *J Bacteriol* **2008**, *190*, doi:10.1128/JB.01402-07.

45. Cui, Z.; Shen, W.; Wang, Z.; Zhang, H.; Me, R.; Wang, Y.; Zeng, L.; Zhu, Y.; Qin, J.; He, P.; et al. Complete Genome Sequence of Klebsiella Pneumoniae Phage JD001. *J Virol* **2012**, *86*, doi:10.1128/jvi.02435-12.

46. Leblanc, C.; Caumont-Sarcos, A.; Comeau, A.M.; Krisch, H.M. Isolation and Genomic Characterization of the First Phage Infecting Iodobacteria: ΦPLPE, a Myovirus Having a Novel Set of Features. *Environ Microbiol Rep* **2009**, *1*, doi:10.1111/j.1758-2229.2009.00055.x.

47. Kazantseva, O.A.; Piligrimova, E.G.; Shadrin, A.M. VB_BcM_Sam46 and VB_BcM_Sam112, Members of a New Bacteriophage Genus with Unusual Small Terminase Structure. *Sci Rep* **2021**, *11*, doi:10.1038/s41598-021-91289-x.

48. Alonso, J.C.; Lüder, G.; Stiege, A.C.; Chai, S.; Weise, F.; Trautner, T.A. The Complete Nucleotide Sequence and Functional Organization of Bacillus Subtilis Bacteriophage SPP1. *Gene* **1997**, *204*, doi:10.1016/S0378-1119(97)00547-7.

49. Daniel, A.; Bonnen, P.E.; Fischetti, V.A. First Complete Genome Sequence of Two Staphylococcus Epidermidis Bacteriophages. *J Bacteriol* **2007**, *189*, doi:10.1128/JB.01637-06.

50. Piligrimova, E.G.; Kazantseva, O.A.; Nikulin, N.A.; Shadrin, A.M. Bacillus Phage VB_BtS_b83 Previously Designated as a Plasmid May Represent a New Siphoviridae Genus. *Viruses* **2019**, *11*, doi:10.3390/v11070624.

51. Simpson, A.A.; Tao, Y.; Leiman, P.G.; Badasso, M.O.; He, Y.; Jardine, P.J.; Olson, N.H.; Morais, M.C.; Grimes, S.; Anderson, D.L.; et al. Structure of the Bacteriophage Φ29 DNA Packaging Motor. *Nature* **2000**, *408*, doi:10.1038/35047129.

52. Morais, M.C.; Koti, J.S.; Bowman, V.D.; Reyes-Aldrete, E.; Anderson, D.L.; Rossmann, M.G. Defining Molecular and Domain Boundaries in the Bacteriophage Φ29 DNA Packaging Motor. *Structure* **2008**, *16*, doi:10.1016/j.str.2008.05.010.

53. Longás, E.; Villar, L.; Lázaro, J.M.; de Vega, M.; Salas, M. Phage Φ29 and Nf Terminal Protein-Priming Domain Specifies the Internal Template Nucleotide to Initiate DNA Replication. *Proc Natl Acad Sci U S A* **2008**, *105*, doi:10.1073/pnas.0809882105.
